# Evaluation of 3-Point and 4-Point Bending Tests for Tensile Strength Assessment of GFRP Bars

**DOI:** 10.3390/ma17215261

**Published:** 2024-10-29

**Authors:** Philip Prakash Lochan, Maria Anna Polak

**Affiliations:** Department of Civil and Environmental Engineering, University of Waterloo, 200 University Ave. West, Waterloo, ON N2L 3G1, Canada; pplochan@uwaterloo.ca

**Keywords:** glass fiber reinforced polymer bars, tensile strength, modulus of rupture, testing, 3-point bending, 4-point bending, Weibull weakest link model

## Abstract

Glass Fiber Reinforced Polymer (GFRP) bars are used as reinforcement for structural concrete, especially in cases where corrosion of traditional steel reinforcement is a problem. The tensile strength of these reinforcing bars is the primary characteristic on which the design of concrete members reinforced with GFRP bars relies. Determination of the tensile strength of the bars using a direct tensile test is a time and resource-intensive task and therefore is not routinely conducted for quality control. The tensile strength can also be measured from flexure tests, which are much simpler than direct tensile tests, and use appropriate correlation formulations. In this paper, the applicability of flexure testing for the identification of bars’ tensile strength is investigated by conducting and analyzing both 3-point and 4-point flexure testing. The correlation formulations are presented that allow the determination of tensile strength from the modulus of rupture. The Weibull weakest link model and the assumption of the same flaw distribution in tensile and flexure tests is adopted. The results of the 3-point and the 4-point bending are presented and compared. Comparisons are also conducted to select direct tensile test results. The work shows that 3-point and 4-point bending tests provide very similar results, with the difference between the results being 2% to 10%, suggesting both tests can be used for tensile strength determination of GFRP bars.

## 1. Introduction

Glass fiber reinforced polymer (GFRP) bars have been gaining popularity as reinforcement for structural concrete, especially in cases where corrosion of traditional steel reinforcements poses a durability problem. Examples include the construction of reinforced concrete using seawater [1] and in the areas prone to problems with corrosion of steel reinforcement, like coastal regions or regions in northern climates with the need for excessive use of deicing salts on highways [2,3]. GFRPs are used as a substitute for steel reinforcements; however, their mechanical behavior and interaction with concrete are different. GFRP bars are strong in tension (often more than 1000 MPa), but they fail by brittle rupture, contrary to steel which yields and has a lot of post-peak load ductility. All types of concrete reinforcements must provide resistance to tensile stresses applied to cross-sections of concrete members and GFRP bars’ tensile strength is their primary mechanical property.

Direct tensile testing of GFRP bars is time and laboratory-intensive and, as a result, usually only performed by manufacturers. Adequate specimen preparation is required with appropriate anchorages at the ends of the specimens [4,5,6]. The specimens are long and heavy and require long and high-capacity testing frames. For large diameter bars the specimens can be over 2.5 m long and require 1000 kN testing frames; therefore, tensile strength cannot be easily determined for such bars. This research describes alternative testing protocols for the determination of the tensile strength of GFRP reinforcing bars, which can be performed using low-capacity testing frames and the appropriate computer analysis.

Because GFRPs are brittle, flexural testing can be used for their tensile strength characterization. Research on the application of bending tests for tensile strength identification was conducted by D’Antino and Pisani [7] who used bars with a full circular cross-section tested in flexure. In contrast, Hosseini et al. [8] used bars cut longitudinally with half cross-sections, which were tested in bending and the obtained results showed conservative estimations of the tensile strengths.

The 3-point bending of GFRP bars was investigated by [4,5] by using a probabilistic approach and the Weibull theory. In this paper, the work is extended to show the procedures and the results for 4-point bending tests, which differ from 3-point bending by having a larger volume of the bar subjected to tension. For both types of tests, the specimens need to be cut longitudinally to create semicircular cross-sections to ensure the tensile failure of the specimens when loaded in flexure. After completing flexure tests, the calculations must be conducted to correlate rupture modulus (tensile strength in bending) to the tensile strength of the bar. The tension-initiated failure stress, or modulus of rupture, depends on the specimen’s geometry, loading and boundary conditions, and size [9]. The modulus of rupture can be correlated to the direct tensile strength using the theory of Weibull’s weakest link [10,11,12] and the concept of effective volumes subjected to tensile stresses. The effective volumes depend on the type of loading on the specimens and are different for 3-point and 4-point bending tests. As the effective volume in tension for the 4-point bending test is larger than for the 3-point bending test it could be postulated that the 4-point test is more accurate. Also, the 3-point bending test creates tensile failure at one specific point in the cross-section directly under the load application, however, the 4-point bending test subjects several points between load applications to the same tension, which can potentially create a more accurate prediction of strength. Therefore, in this study, a comparison between results from both types of testing, including some tensile testing, are provided and discussed. The work includes testing of a variety of bar diameters, namely 8 mm (M8), 13 mm (M13), 16 mm (M15), 20 mm (M20), 25 mm (M25), and 32 mm (M32).

## 2. Test Procedures and Specimens

**Figure 1:**
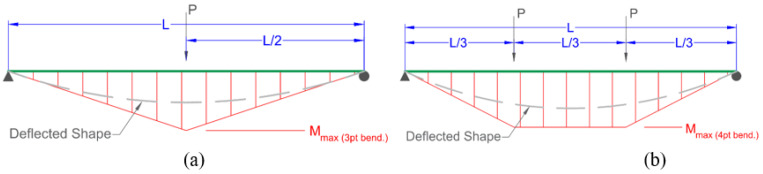
Schematic of flexural testing with deflected shapes and bending moment diagrams (**a**) 3-point, (**b**) 4-point.

Flexural testing performed in this work includes 3-point and 4-point bending tests as per the schematic in Figure 1. The testing followed ASTM D4476 [13] and CSA S807-10 [14]. For each bar size, 10 specimens were tested. The flexure tests involved bending semicircular bars until failure. The preparation of the specimens involved cutting the bars to the required length and along their length in order to ensure tensile failure occurred before compression failure. The longitudinal cutting was conducted using waterjet cutting. During testing the applied loading of the machine’s crosshead was recorded. The load corresponding to the failure of fibers in tension was determined. This cracking load of the bar is slightly lower than the ultimate load as after the first cracking, the specimen still deforms further until both fibers in tension and compression fail. From the cracking load, the rupture modulus was calculated using the procedures described later in this paper.

**Figure 2:**
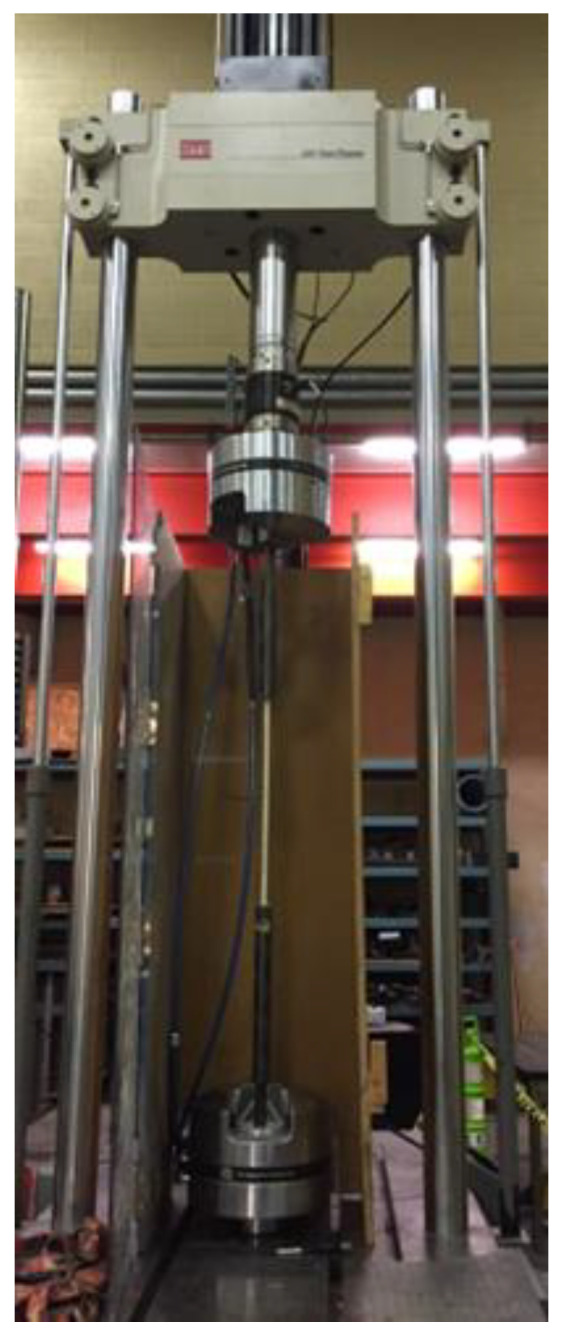
Tensile testing of GFRP bars.

Selected direct tensile testing of bars M8, M13, and M15 was also conducted. This was based on ASTM D7205 [15]. These tests allowed for direct comparisons between correlated tensile strength obtained from bending tests and the results of direct tension tests. The largest bars we could test were M15 due to the equipment and laboratory constraints. Larger bars require higher capacity and higher length test frames, as the specimen strengths and length get larger with its diameter. The test involves casting the ends of the specimens in large DOM steel tubes, which allows for the specimens to be gripped and the bars to be pulled with uniform stress across the cross-section, ensuring tensile failure of the bars and not anchorage failure (Lochan and Polak 2022 [4]). The specimens’ length was 1320 mm for M8, 1520 mm for M13, and 1640 mm for M15. Four specimens were tested for M8 and five specimens for M13 and M15 bars. A summary of these test results is shown in Table 1. A typical specimen in the test frame (MTS 320, 500 kN capacity) is shown in Figure 2.

All specimens used in the testing were provided by Fiberline Composites (Kitchener, ON, Canada). They were straight, ribbed-surfaced GFRP bars (fiber content 88% by weight), with the diameters and cross-sectional areas listed in Table 2.

**Figure 3:**
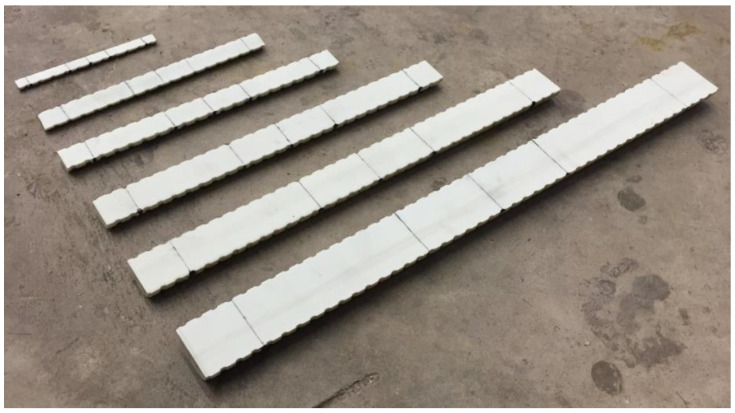
GFRP specimens for flexure tests. The lines show the location of applied loading.

The clear span length and total length of specimens are reported in Table 2. The lengths of the specimens were cut to specifications listed in ASTM D4476 [13]. The total length of a specimen was 16 to 24 times the diameter, plus a 10% overhang on each side (Table 2). The specimens were longitudinally cut resulting in almost semicircular cross-sections (due to blade thickness). Each specimen’s cross-section was measured individually for further analysis. Figure 3 shows one specimen of each bar size cut to length and longitudinally, indicating the placement of applied loading for either 3-point or 4-point bending tests.

## 3. Testing Equipment

**Figure 4:**
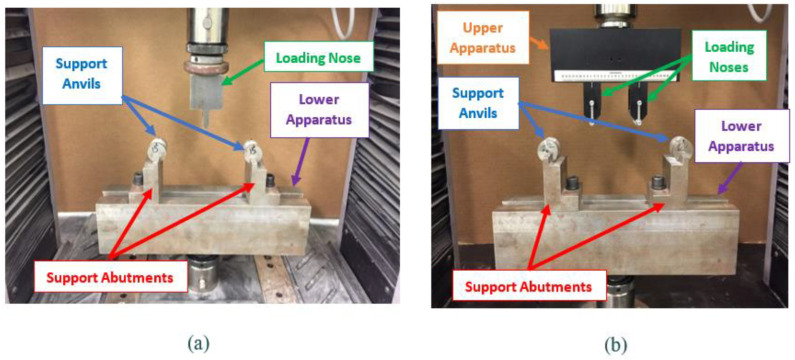
Flexure testing equipment used in (**a**) 3-point bending tests and (**b**) 4-point bending tests.

In order to complete the flexure tests, a fixture similar to ASTM D4476 [13] attached to a low-capacity (100 kN) MTS electromechanical testing frame was required. The specimen is held in place on two supports, which rest on support abutments that can move along the lower apparatus. The top fixture for a 3-point bending test is a single loading nose that exerts a load onto the GFRP specimen at midspan, as seen in Figure 4a. For a 4-point bending test, the top fixture is similar to the lower apparatus, where there are two load noses that can move along the length into the correct placement of the applied load. This test setup is shown in Figure 4b.

## 4. Test Observations and Results

**Figure 5:**
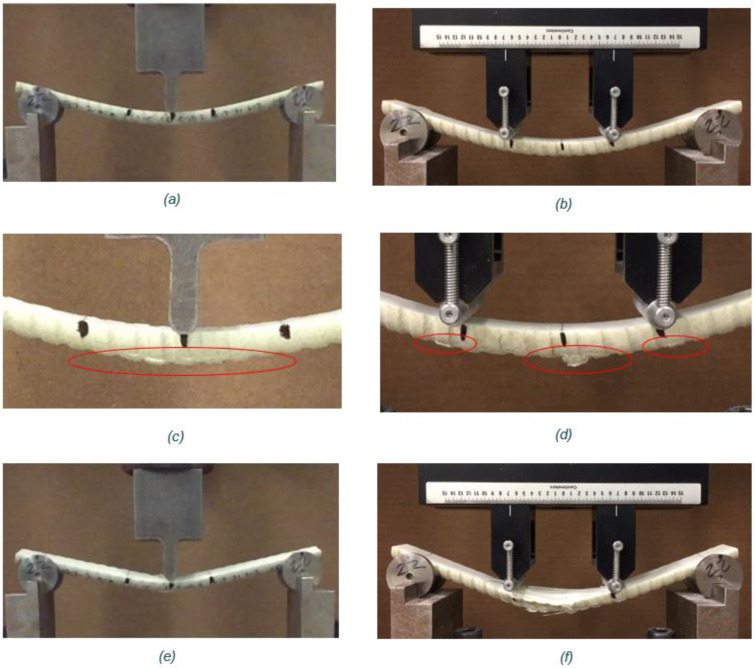
Example of the behavior of a M20 bar under 3-point and 4-point bending: (**a**) 2-point before failure, (**b**) 4-point before failure, (**c**) Close-up view of tensile fiber rupture in a 3-point bending, (**d**) Close-up view of tensile fiber rupture in a 4-point bending, (red circles show rupture zones) (**e**) After failure in 3-point bending, and (**f**) After failure in 4-point bending.

**Figure 6:**
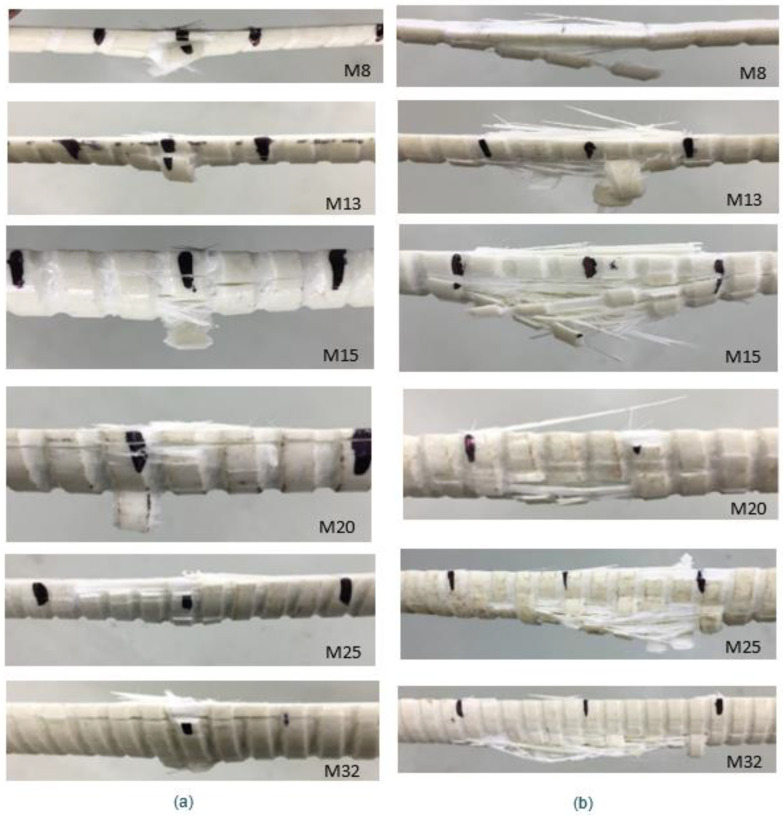
Tensile face of specimen after testing in (**a**) 3-point bending and (**b**) 4-point bending.

The close-up photos of the failed tension surfaces in the tested specimens are shown in Figure 5. It is visible that in 3 point-testing the point under the loading nose cracked (Figure 5c,e), while in 4 point-bending tests, the whole length between load applications was cracked at failure (Figure 5d,f). Figure 6 shows the failure of specimens of each size after testing, where it is evident that the tensile face has ruptured fibers. For the 3-point bending, only one location under the applied load ruptured. However, for the 4-point bending the entire bottom line of fibers (line between load applications) of the specimen ruptured at failure.

**Figure 7:**
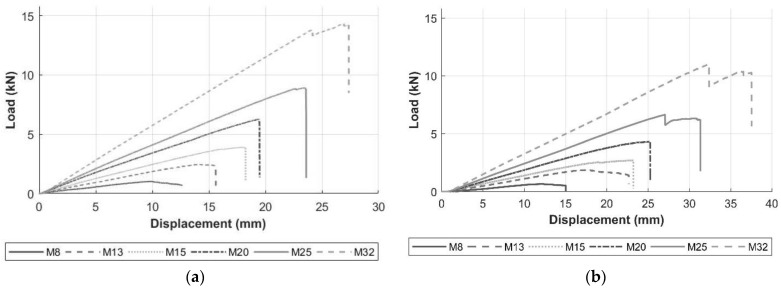
Examples of load-displacement plots of GFRP flexure specimens in (**a**) 3-point bending and (**b**) 4-point bending.

The examples of load-displacement plots, recorded during the tests, are shown in Figure 7 for all bar diameters. For the 3-point bending test, the graph shows the force (load) exerted by the loading nose versus central displacement recorded by the testing equipment. For the 4-point bending test, the results show the force versus central displacement (note the full load on the bar was 2× force). Similar patterns are noted for both types of flexure tests. All specimens show linear trends, where the response is more linear as the bar size increases. The shape of the load-displacement plot for each specimen becomes non-linear as the ultimate load is approached. Table 3 displays the ultimate loads and the corresponding deflection for each bar size from the respective flexure test.

## 5. Determination of Rupture Modulus

Flexural testing allows us to determine the rupture modulus of the bars. The first step in this process is finding the cracking load corresponding to the first rupture of tensile fibers under bending loads. This load must be identified from the load-displacement laboratory data and corresponds to the point in the graph where there is significant slope change, which signifies fiber breakage in the specimen. The automated and objective method to determine this point was outlined by Lochan and Polak [4] and it involves numerical differentiation of the experimental load-displacement response. The procedure starts with smoothing the laboratory data [16] to eliminate the “noise” in the response. A double exponential filtering method was used for this purpose [17].

**Figure 8:**
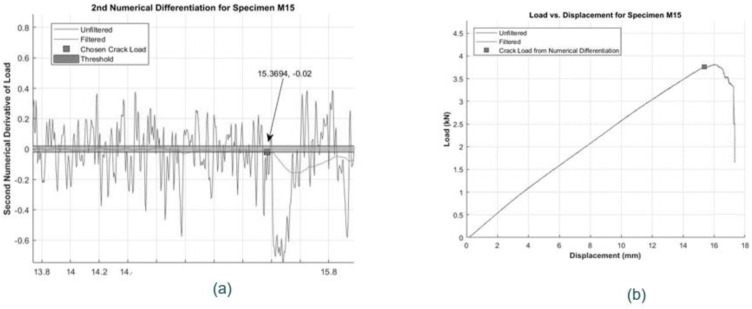
(**a**) Zoomed-in view of chosen cracking load from 2nd numerical derivative of load-displacement plot for M15 specimen, and (**b**) Example of a chosen Cracking Load on Load-Displacement Plot for M15 Specimen.

The change in the slope of the laboratory data is found by taking the second derivative of a smoothed data set. For the constant slope of the load-displacement plots, the second derivative is theoretically equal to zero, and for real data it is close to zero. This trend is determined by specifying a user-defined threshold around zero value, which in this case is determined to be ±0.02 [4], and checking whether the smoothed second derivative falls within this threshold. When the second derivative deviates from the zero threshold it indicates the cracking load. Figure 8a shows the selected data point for the cracking load within the threshold, while Figure 8b shows the same chosen point plotted on the corresponding load-displacement graph.

**Figure 9:**
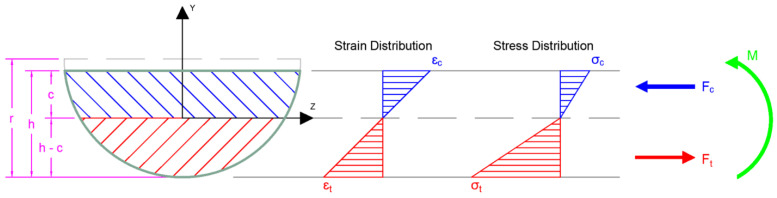
Cross-section and distribution of bending strains and stresses of GFRP flexure specimen.

The rupture modus is determined from the cracking load using the force and moment equilibrium on the semicircular cross-sections (each cross-section was measured for exact dimensions) and the stress-strain relationships in tension and compression (Figure 9, [4]). The linear bi-modular behavior of GFRP material, which differs in tension and compression [5] was adopted in the calculations. Glass fiber reinforced polymers have different stiffnesses in tension and compression, with the tensile stiffness being higher. In the presented results, the ratio of the moduli, namely $n=\frac{E_{t}}{E_{c}}$ plays the role. The suggested values of this ratio, based on the literature [18,19], are between 1.2 and 1.25. Arczewska et al. [5] obtained a ratio of 1.2 and in the previous work [4] suggested using the ratio of 1.2, therefore this is used in the work presented herein. It should be noted that when a lower ratio n $=\frac{E_{t}}{E_{c}}$ is used the results are more conservative (the correlated tensile strength is lower). The calculations of moduli of rupture required integration through the depth of the specimens, which were performed using a numerical layered approach with each specimen discretized by 100 layers of equal thickness [4,17].

Table 4 presents the average cracking load and rupture modulus for each specimen size per flexure test. To assure better accuracy, specimens of each size with the lowest and highest rupture moduli were considered as “outliers” and were not included in the calculations. Please note that for each bar size, 10 specimens were tested.

Based on Table 4, it is evident that the ratios between the rupture moduli obtained from the 3-point and the 4-point bending tests are between 1 and 1.1, indicating that the two types of flexure tests yield similar results. However, except for M8 bars, the general trend suggests that the 4-point bending results in a slightly lower rupture modulus than the 3-point bending. This is consistent with the rupture of brittle material, which depends on the amount of weak links in the tension zone. The volume subjected to tension in 4-point bending is much larger than in 3-point bending, making it more likely to encounter the weakest point that initiates cracking. Therefore, it is expected that the moduli of rupture should be smaller for 4-point bending than for 3-point bending.

## 6. Weibull Strength Scaling Model

The strength scaling between the direct tension and the flexure tests was conducted using the Weibull weakest link model [10,11,12]. According to the Weibull model, the material’s strength is defined by the number of flaws present in the volume of the material, which is referred to as its “weakest link”. The assumption is also made that the distribution of flaws in the volume is uniform and the failure distribution follows a (two-parameter) Weibull distribution, with the probability of material failure represented by Equation (1) as follows:

(1) $$P_{f}=1-\exp\left(-\int_{V}{\left(\frac{\sigma -\sigma_{u}}{\sigma_{o}}\right)}^{m}dV\right)$$

- V represents the volume of the specimen;
- σ represents the applied stress;
- $\sigma_{u}$ represents the zero-strength stress where no failure occurs below this stress (which is usually assumed to be zero);
- $\sigma_{o}$ is the normalizing factor (the scale parameter);
- m is the Weibull modulus (shape parameter).

Assuming that the probability of failure of samples placed under direct tension and under bending tests are the same, and $\sigma_{u}=0$ the following is obtained:

(2) $$\int_{V_{t}}{\left(\frac{\sigma_{t}}{\sigma_{o}}\right)}^{m}dV_{t}=\int_{V_{b}}{\left(\frac{\sigma_{b}}{\sigma_{o}}\right)}^{m}dV_{b}$$

From Equation (2) the ratio between the modulus of rupture and tensile strength can be calculated as follows:

(3) $$\frac{\sigma_{r}}{\sigma_{t}}={\left(\frac{V_{Et}}{V_{Eb}}\right)}^{\frac{1}{m}}$$

where

- $\sigma_{t}$ and $\sigma_{b}$ are the tensile stresses in the direct tension and flexure test, respectively;
- $\sigma_{r}$ is the modulus of rupture;
- $V_{t}$ and $V_{b}$ are the volumes of a specimen experiencing tensile stress subjected to a uniaxial direct tensile load and a flexural load, respectively;
- $V_{Et}$ and $V_{Eb}$ refer to the effective volumes experiencing tensile stress in the uniaxial direct tensile test and a flexural test. $V_{Eb}$ and $V_{Et}$.

The procedure requires calculations of the effective volumes in tension in direct tensile tests and flexure tests. The left-hand side of Equation (2) represents direct tension, and the right-hand side represents bending. In the case of direct tension, $\sigma_{t}$ is constant throughout the volume of the specimen and, at rupture, it is equal to the tensile strength. The total volume V is equal to the effective volume in the direct tension test $V_{Et}$.

(4) $$\int_{V}{\left(\frac{\sigma_{t}}{\sigma_{o}}\right)}^{m}dV_{t}={\left(\frac{\sigma_{t}}{\sigma_{o}}\right)}^{m}\int_{V}dV_{t}={\left(\frac{\sigma_{t}}{\sigma_{o}}\right)}^{m}\times V_{Et}$$

The effective volume of tensile stress $V_{Et}$ is the entire volume of a specimen

(5) $$V_{Et}=A\times L$$

(6) $$A=r\left(r\times \cos^{-1}\left(\frac{d}{r}\right)-d\times \sqrt{1-\frac{d^{2}}{r^{2}}}\right)$$

where

- d=r−h;
- L is the length of the flexure specimen between the supports;
- h is the height of the specimen (Figure 9);
- r is the original radius of the specimen (Figure 9).

For the flexure test the volume that is in tension depends on the loading conditions and it is different for 3-point and 4-point bending. In general, $\sigma_{b}=f\left(x,y\right)\sigma_{r}$, where $\sigma_{r}$ is the modulus of rupture and $f\left(x,y\right)$ represents the distribution of tensile stresses within the specimen.

(7) $$\int_{V_{b}}{\left(\frac{\sigma_{b}\left(x,y\right)}{\sigma_{o}}\right)}^{m}dV_{b}={\left(\frac{\sigma_{r}}{\sigma_{0}}\right)}^{m}\int_{V_{b}}{f\left(x,y\right)}^{m}dV_{b}={\left(\frac{\sigma_{r}}{\sigma_{0}}\right)}^{m}V_{Eb}$$

(8) $$V_{Eb}=\int_{V_{b}}{f\left(x,y\right)}^{m}dV_{b}=\int_{V_{b}}{\left(\frac{\sigma_{b}\left(x,y\right)}{\sigma_{r}}\right)}^{m}dV_{b}$$

Calculation of effective volumes in tension in the flexure tests $V_{Eb}$ required specifying the function $f\left(x,y\right)=\frac{\sigma_{b}\left(x,y\right)}{\sigma_{r}}$. The tensile stress along the length and the height of the specimen subjected to bending $\sigma_{b}\left(x,y\right)$ is represented in Equations (9) and (10) for 3-point bending, and by Equations (11)–(13) for 4-point bending. These equations correspond to the bending moment diagrams for the respective flexural loading and the variation along the height (Figure 1). It is also important to note the tensile stress distribution along the height of the cross-section varies linearly.

For the 3-point bending tests, the equation is as follows:

(9) $$\sigma_{b}\left(x,y\right)=-\left(\frac{y}{h-c}\right)\left(\frac{2x}{L}\right)\sigma_{r}\;\mathrm{for}\;0<x<\frac{L}{2}$$

(10) $$\sigma_{b}\left(x,y\right)=-\left(\frac{y}{h-c}\right)\left(\frac{2\left(L-x\right)}{L}\right)\sigma_{r}\;\mathrm{for}\;\frac{L}{2}<x<L$$

For the 4-point bending tests, the equation is as follows:

(11) $$\sigma_{b}\left(x,y\right)=-\left(\frac{y}{h-c}\right)\sigma_{r}\;\mathrm{for}\;\frac{L}{3}<x<\frac{2L}{3}$$

(12) $$\sigma_{b}\left(x,y\right)=-\left(\frac{y}{h-c}\right)\left(\frac{3x}{L}\right)\sigma_{r}\;\mathrm{for}\;0<x<\frac{L}{3}$$

(13) $$\sigma_{b}\left(x,y\right)=-\left(\frac{y}{h-c}\right)\left(\frac{3(L-x)}{L}\right)\sigma_{r}\;\mathrm{for}\;\frac{2L}{3}<x<L$$

Inserting Equations (9)–(13) into Equation (8) results in the following equations used for the calculation of effective volumes.

For the 3-point bending test, the equation is as follows:

(14) $$V_{Eb,3}=2\int_{V_{b}}{\left(-\left(\frac{y}{h-c}\right)\left(\frac{2x}{L}\right)\right)}^{m}dV_{b}=\left(-\frac{2^{m+1}}{(\left(h-c\right){L)}^{m}}\right)\int_{0}^{L/2}\int_{0}^{-(h-c)}\int_{-\sqrt{r^{2}-{\left(y-\left(r-\left(h-c\right)\right)\right)}^{2}}}^{\sqrt{r^{2}-{\left(y-\left(r-\left(h-c\right)\right)\right)}^{2}}}y^{m}x^{m}dzdydx$$

For the 4-point bending test, the equation is as follows:

(15) $$V_{Eb,4}=\left\{2\int_{0}^{\frac{L}{6}}\int_{A_{t}}{\left(-\left(\frac{y}{h-c}\right)\right)}^{m}dA_{t}dx+2\int_{0}^{\frac{L}{3}}\int_{A_{t}}{\left(-\left(\frac{y}{h-c}\right)\left(\frac{3x}{L}\right)\right)}^{m}dA_{t}dx\right\}=\;\left(\frac{2{\left(-1\right)}^{m}}{{\left(h-c\right)}^{m}}\right)\int_{0}^{\frac{L}{6}}\int_{0}^{-(h-c)}\int_{-\sqrt{r^{2}-{\left(y-\left(r-\left(h-c\right)\right)\right)}^{2}}}^{\sqrt{r^{2}-{\left(y-\left(r-\left(h-c\right)\right)\right)}^{2}}}y^{m}dzdydx+\left(\frac{2{\left(-1\right)}^{m}{\left(3\right)}^{m}}{{\left(h-c\right)}^{m}L^{m}}\right)\int_{0}^{\frac{L}{3}}\int_{0}^{-(h-c)}\int_{-\sqrt{r^{2}-{\left(y-\left(r-\left(h-c\right)\right)\right)}^{2}}}^{\sqrt{r^{2}-{\left(y-\left(r-\left(h-c\right)\right)\right)}^{2}}}y^{m}x^{m}dzdydx$$

where

- L is the length of the specimen;
- h is the height of the specimen (Figure 9);
- c is the depth of the neutral axis (Figure 9);
- r is the original radius of the specimen (Figure 9);
- z-coordinate refers to the width of the cross-section;
- y-coordinate refers to the height/depth of the cross-section;
- x-coordinate refers to the length of the cross-section.

**Figure 10:**
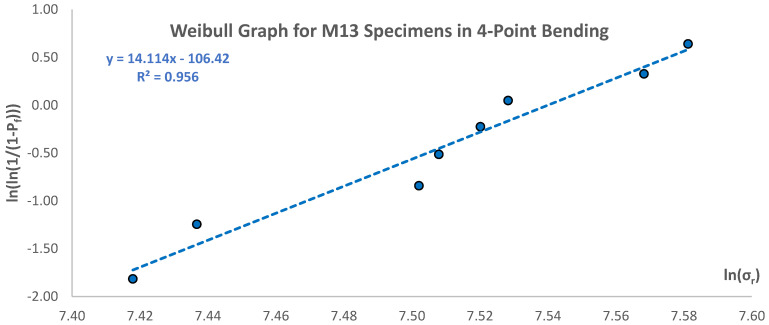
Weibull strength distribution graph for M13 data in 4-point bending (except first and last data points).

In order to use Equations (14) and (15), the Weibull moduli (m) must be determined from testing specimens in flexure, using a Weibull strength distribution graph for each specimen size in the flexure tests [5,17]. A sample Weibull strength distribution graph is shown in Table 5 and plotted in Figure 10. The slope of the established trendline is the value of the Weibull modulus. The same calculations were conducted for all specimens for both types of flexure tests and the results are presented in Table 6.

The Weibull moduli are indicators of the distribution of flaws in the material and the lower modulus indicates more variability in the flaw distribution. The two types of flexure testing resulted in quite different Weibull moduli. The 4-point bending, generally, results in the lower moduli. This is reasonable because in 4-point bending more material is subjected to tensile stresses. There is also a possibility that testing more than 10 samples (as used in this work) would improve the accuracy and allow us to obtain more similar Weibull moduli in both types of testing.

Knowing the Weibull moduli allows evaluations of the effective volumes using Equations (14) (3-point bending) and (15) (4-point bending) which were completed in Maple software [20]. The average effective volumes are displayed in Table 7. The effective volumes in flexure were calculated using corresponding Weibull moduli from Table 6. It should also be noted that the calculated volumes for direct tension are slightly different for the 3-point and the 4-point bending tests because these are averages for specimens in each batch of testing and are based on actual measurements taken for each specimen.

The effective volume is higher for the 4-point bending specimens compared to the 3-point bending specimens due to the different stress distributions throughout the volume of the specimens.

## 7. Correlated Tensile Strength and Discussion

**Figure 11:**
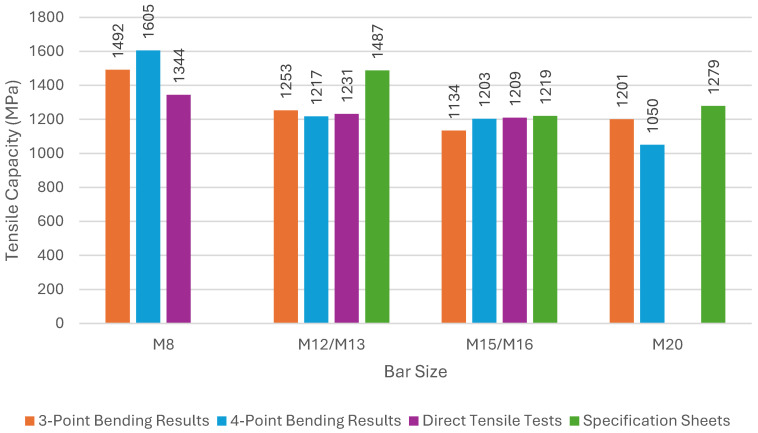
Comparison of tensile capacities from sources within research.

The correlated tensile capacity can be evaluated by dividing the rupture moduli by the calculated tensile ratio. Table 8 displays the calculated tensile capacity ratios and correlated tensile capacities for both the 3-point and the 4-point bending tests. Also shown in Table 8 are the tensile capacities of the bars obtained through direct tensile testing completed as part of this research program, along with the tensile capacities provided by the manufacturer. Figure 11 shows a summary of this information, comparing the correlated tensile capacities from the 3-point and the 4-point bending results to the tensile capacities from testing and manufacturers.

The differences between the correlated (scaled) tensile capacities from both types of flexure tests are small. The maximum difference is for M20 which is 13%. Based on this test program it seems that both 3-point and 4-point bending tests can yield acceptable results.

The differences between the correlated tensile capacities and the tensile capacities from direct tensile testing do not exceed 7%, with the exception of the M8 specimens where the difference is 18%. This is an indication that both flexure tests yield accurate tensile capacities. However, the differences are lower for the 4-point bending tests compared to the 3-point bending, except with the M8 specimens.

From the presented testing and analysis several comments can be offered. The Weibull moduli obtained from the 4-point bending tests are generally lower than from the 3-point bending tests. This could be because the 4-point bending test subjects a larger volume of the specimen to tension compared to the 3-point bending test. Weibull modulus is an indication of the uniformity of flaws and in more volume, there are more options for flaws. Also, the cracking process in 3-point bending is characterized by quite distinct cracking of a point under the load application, as shown in Figure 4 and Figure 5. The 4-point bending results in progressive cracking between the load application points and detection of the actual cracking load (purpose of this work) might be difficult, even with the application of the automated cracking load detection method used in this work [4].

The 3-point bending test is slightly easier to conduct and analyze. The cracking and failure in 3-point bending are well defined by one point on the specimen, under the point load. This can be easier identified than in the 4-point bending that results in the cracking of several locations between the loading noses. To improve accuracy in detecting flaw distributions in the 3-point bending test, more specimens can be tested, e.g., 20 versus the 10 that were tested in this program. This test is very fast and simple and testing requires a few minutes per specimen.

The largest discrepancy in direct tension test results and the scaled tension strength from bending is for the M8 bars. This is true for both the 3-point and the 4-point bending. It should be noted that the ASTM D4476 standard [13] is for bars larger than ½ inch in diameter and M8 bars do not fall under this standard category. These are very small bars, and their strength could be expected to be larger than for larger bars and this was the case for both scaled tensile strength and direct tensile strength. The failure modes in flexure indicate the expected behavior. However, determination of the cracking loads for these small bars was difficult due to the nonlinear behavior of the bars under flexure loads close to failure (Figure 6). This could have contributed to high-scaled tensile strengths for both flexure tests for M8 bars when compared to the direct tensile testing strength (Table 8). On the other hand, the values obtained from direct tensile testing could be slightly reduced due to potential eccentricity of loading and/or misalignments caused by errors in placing the bars in the tensile frame. The bars are slender and with heavy steel DOM tubes at the ends are not easy to keep perfectly concentric.

Another observation for the M8 bars is that when looking at the Weibull moduli obtained from both types of flexure test, they are rather high (20.5 and 18), indicating a uniform distribution of flaws and high quality of bars, which could suggest that the tensile scaled capacities are correct. They are also similar for both types of flexure testing, indicating consistency in the results.

## 8. Conclusions

The results of flexure testing and the corresponding calculations required to determine the scaled tensile strength of GFRP bars from flexure tests are presented in the paper. Glass fiber reinforced polymer bars of different diameters were tested in two configurations in the flexure test, namely 3-point and 4-point bending. The primary purpose of this work was to determine if using one of these flexure testing configurations would prove to be more beneficial than the other and if potentially both testing types could be used for the determination of the tensile strength of GFRP bars. Corresponding direct tensile tests were also conducted on selected bars, for comparison purposes.

It should be noted that direct tensile testing should be considered the best indicator for strength of GFRP bars during the process of product identification and testing performed by manufacturers. However, for quality control, flexure testing is faster and more realistic for routine performance, serving as a good indicator of tensile strength for quality control of the bars used in construction.

Based on this work the following conclusions can be offered:

1. Using a flexure test to determine the tensile capacity of GFRP bars is easy, convenient, and fast when compared to the uniaxial direct tensile test. Cutting the GFRP bars longitudinally in half ensures that tensile failure occurs first and that the corresponding rupture modulus can be correlated to the tensile capacity.
2. Both 3-point and 4-point flexure tests provide reliable results in terms of correlating rupture moduli to the tensile strength. The 4-point bending test can potentially be better in detecting flaw distributions within the specimens because more volume is subject to tension during testing when compared to the 3-point bending. However, identifying cracking loads can be more difficult for the test where several points between the loading points are cracking simultaneously.
3. Both tests are good tests for the purpose of tensile strength identification; however, the 3-point bending can be considered an easier option for flexural testing of GFRP bars to determine their tensile strength.

## Figures and Tables

**Table 1:** Summary of Uniaxial Direct Tensile Testing Results.

Parameter	Values	M8	M13	M15
Maximum Force $F_{max}\;(N)$	Avg.	67,574.24	163,423.88	243,099.93
	SD	1661.01	3112.16	4502.52
	COV	0.02	0.02	0.02
Maximum Displacement ${∆}_{F_{max}}\;(mm)$	Avg.	12.08	20.10	24.52
	SD	0.70	1.25	1.41
	COV	0.06	0.06	0.06

**Table 2:** GFRP dimensions (cross-sectional properties per supplier information).

Bar Size	Core Diameter (mm)	Exterior Diameter (mm)	Cross-Section Area (mm^2^)	Length Flexure Specimens (mm)		Length Tensile Specimens (mm)	
				Clear Span L	Total L_T_	Free Length L	Total L_T_
M8	8	9	50.3	80	96	320	1320
M13	13	14.5	132	130	156	520	1520
M15	16	18	201	160	192	640	1640
M20	20	22	314	200	240	-	-
M25	25	27	491	250	300	-	-
M32	32	34	804	320	384	-	-

**Table 3:** Summary of maximum loads $F_{max}$ and corresponding midspan deflections ${∆}_{F_{max}}$.

			M8	M13	M15	M20	M25	M32
3-Point Bending	$F_{max}\;(kN)$	Avg.	0.90	2.54	3.96	6.18	8.76	14.13
		SD	0.11	0.12	0.08	0.25	0.36	0.51
		COV	0.12	0.05	0.02	0.04	0.04	0.04
	${∆}_{F_{max}}\;(mm)$	Avg.	9.79	14.24	17.31	20.04	22.30	26.65
		SD	0.65	0.32	0.71	0.56	1.00	1.52
		COV	0.07	0.02	0.04	0.03	0.04	0.06
4-Point Bending	$F_{max}\;(kN)$	Avg.	0.69	1.81	2.78	4.50	6.60	10.63
		SD	0.06	0.10	0.11	0.14	0.29	1.40
		COV	0.09	0.06	0.04	0.03	0.04	0.13
	${∆}_{F_{max}}\;(mm)$	Avg.	13.44	19.97	24.11	28.96	34.08	38.31
		SD	0.89	0.59	1.69	0.93	1.51	2.76
		COV	0.07	0.03	0.07	0.03	0.04	0.07

SD = standard deviation. COV = coefficient of variation.

**Table 4:** Summary of Flexure Tests.

			M8	M13	M15	M20	M25	M32
3-Point Bending	$P_{cr}\;(N)$	Avg.	912.74	2366.13	3801.58	5755.94	8361.49	13,365.87
		SD	101.20	116.31	111.60	201.42	346.45	175.30
		COV	0.11	0.05	0.03	0.03	0.04	0.01
	$\sigma_{r}\;(MPa)$	Avg.	2218.59	1863.53	1857.35	1751.24	1666.20	1589.79
		SD	86.77	71.08	97.53	63.14	53.03	59.46
		COV	0.04	0.04	0.05	0.04	0.03	0.04
4-Point Bending	$P_{cr}\;(N)$	Avg.	647.83	1713.64	2517.43	3868.00	6012.09	10,099.69
		SD	53.18	126.87	168.99	225.71	326.19	206.89
		COV	0.08	0.07	0.07	0.06	0.05	0.02
	$\sigma_{r}\;(MPa)$	Avg.	2226.84	1804.38	1692.40	1590.64	1638.89	1577.82
		SD	98.63	101.92	80.13	95.51	76.30	56.53
		COV	0.04	0.06	0.05	0.06	0.05	0.04
$\frac{\sigma_{r_{3-Pt\;Bend}}}{\sigma_{r_{4-Pt\;Bend}}}$			1.00	1.03	1.10	1.10	1.02	1.01

$P_{cr}=cracking\;load,\;\sigma_{r}=rupture\;modulus$, SD = standard deviation, COV = coefficient of variation.

**Table 5:** Data for Weibull strength distribution graph for M13 data in 4-point bending (except first and last data points).

Rank, i	Test No.	Specimen No.	Rupture Modulus, σ_r_ (MPa)	$P_{f}=\frac{\left(i-0.5\right)}{n}$	$x=ln(\sigma_{r})$	$y=\ln\left(\ln\left(\frac{1}{1-P_{f}}\right)\right)$
1	78	M13-16	1665.56	0.15	7.42	−1.82
2	74	M13-4	1697.21	0.25	7.44	−1.25
3	75	M13-8	1811.85	0.35	7.50	−0.84
4	77	M13-17	1822.47	0.45	7.51	−0.51
5	79	M13-5	1844.93	0.55	7.52	−0.23
6	81	M13-9	1860.01	0.65	7.53	0.05
7	73	M13-7	1935.90	0.75	7.57	0.33
8	82	M13-19	1961.16	0.85	7.58	0.64
m = 14.11			b = −106.42		R^2^ = 0.9560	

**Table 6:** Weibull modulus for each specimen size per flexure test.

Test	M8	M13	M15	M20	M25	M32
3-point	20.5	20.5	15	22	23.5	21
4-point	18	14	17	13	15.5	21.5

**Table 7:** Summary of effective volume of maximum tensile stress.

			M8	M13	M15	M20	M25	M32
3-Point Bending	V_eb3_ (mm^3^)	Avg.	0.49	2.29	9.17	7.42	12.24	33.92
		SD	0.03	0.07	0.20	0.09	0.29	0.77
		COV	0.07	0.03	0.02	0.01	0.02	0.02
	V_Et_ (mm^3^)	Avg.	1661.63	7780.40	15,018.34	29,758.95	57,388.77	121,885.50
		SD	111.39	207.78	298.37	331.36	1240.64	2541.55
		COV	0.07	0.03	0.02	0.01	0.02	0.02
4-Point Bending	V_eb4_ (mm^3^)	Avg.	4.44	31.41	44.80	133.75	197.61	264.59
		SD	0.16	0.98	0.78	1.62	3.90	5.39
		COV	0.04	0.03	0.02	0.01	0.02	0.02
	V_Et_ (mm^3^)	Avg.	1608.78	7766.74	14,745.19	29,539.18	56,581.33	122,876.59
		SD	55.33	223.10	237.15	330.19	1028.92	2300.82
		COV	0.03	0.03	0.02	0.01	0.02	0.02

**Table 8:** Summary of calculated tensile capacity ratio and correlated tensile capacity.

			M8	M13	M15	M20	M25	M32
$\frac{\sigma_{b}}{\sigma_{t}}={\left(\frac{V_{Et}}{V_{Eb}}\right)}^{\frac{1}{m}}$	3-Point Bending	Avg.	1.49	1.49	1.64	1.46	1.43	1.48
		SD	0.00	0.00	0.00	0.00	0.00	0.00
		COV	0.00	0.00	0.00	0.00	0.00	0.00
	4-Point Bending	Avg.	1.39	1.48	1.41	1.51	1.44	1.33
		SD	0.00	0.00	0.00	0.00	0.00	0.00
		COV	0.00	0.00	0.00	0.00	0.00	0.00
	% Diff between 3-Point and 4-Point Bending		6.97%	0.31%	15.22%	3.81%	0.53%	10.41%
Scaled Tensile Capacity (MPa)	3-Point Bending	Avg.	1491.58	1253.30	1133.97	1201.04	1162.82	1076.51
		SD	58.24	47.75	59.43	43.28	36.97	40.15
		COV	0.04	0.04	0.05	0.04	0.03	0.04
	4-Point Bending	Avg.	1605.19	1217.26	1203.44	1050.14	1137.73	1185.81
		SD	71.13	68.75	57.03	63.04	52.93	42.40
		COV	0.04	0.06	0.05	0.06	0.05	0.04
	% Diff between 3-Point and 4-Point Bending		7.34%	2.92%	5.94%	13.41%	2.18%	9.66%
Tensile Capacity from Direct Tensile Testing (MPa)			1344.35	1231.23	1209.08	-	-	-
% Diff. between 3-Point Bending			10.38%	1.78%	6.41%	-	-	-
% Diff. between 4-Point Bending			17.69%	1.14%	0.47%	-	-	-
Tensile Capacity from Specification Sheets (MPa)			-	1487.40	1219.40	1278.80	-	-
% Diff. between 3-Point Bending			-	17.08%	7.26%	6.27%	-	-
% Diff. between 4-Point Bending			-	19.98%	1.32%	19.64%	-	-

## Data Availability

The original contributions presented in the study are included in the article, further inquiries can be directed to the corresponding author.
